# HemOncAgent: an artificial intelligence system for retrieving structured and narrative oncology knowledge

**DOI:** 10.64898/2026.09.09.26362651

**Published:** 2026-09-11

**Authors:** Andrew J Yang, Hossam A Zaki, Aaron Seto, Taemin Kim, Irbaz Bin Riaz, Andrew Srisuwananukorn, Andrew J Cowan, Peter C Yang, Jeremy L Warner

## Abstract

Oncology knowledge spans structured and unstructured datasets of drugs, regimens, conditions, and identifiers, as well as clinician-authored narratives describing treatment sequencing across disease settings. Answering clinical questions may require accessing both databases and free-text narratives, but existing intelligent retrieval systems favor either relational facts or narrative context. We developed HemOncAgent, an artificial intelligence agent that selects among retrieval tools for the HemOnc knowledge ecosystem: HemOncKB, a curated knowledge graph for pharmacologic relationships, and HemOnc.org , a narrative resource for care pathways. Across structured and narrative benchmarks, HemOncAgent maintained high-fidelity retrieval across question types for which single-source systems showed domain-specific limitations, supporting hybrid tool-based retrieval for more reliable oncology knowledge access.

## Full Text Availability

The license terms selected by the author(s) for this preprint version do not permit archiving in PMC. The full text is available from the preprint server.

